# Disseminated Nocardiosis: A Successful Blind Strategy of Treatment in an HIV Infected Patient

**DOI:** 10.1155/2015/260640

**Published:** 2015-03-02

**Authors:** Ana C. Guerra, Dário Batista, Maria J. Aleixo, Paulo Saraiva, Maria J. Aguas

**Affiliations:** ^1^Infectious Diseases Department, Garcia de Orta Hospital, Avenida Torrado da Silva, 2801-951 Almada, Portugal; ^2^Internal Medicine Department, Garcia de Orta Hospital, Avenida Torrado da Silva, 2801-951 Almada, Portugal; ^3^Neuroradiology Department, Garcia de Orta Hospital, Avenida Torrado da Silva, 2801-951 Almada, Portugal

## Abstract

*Background.* Nocardiosis is a rare disease that mainly affects severely immunocompromised patients. Symptoms are nonspecific and microbiological isolation is difficult, hiding the diagnosis. Treatment should be guided by species and susceptibility testing. *Findings.* We report a clinical case of a disseminated nocardiosis in a patient with HIV and HVB infections. *Interpretation.* Diagnosis should be presumed early and microbiological conditions should be optimized, in order to identify the species and achieve antibiotic susceptibility testing. This is a very important step to choose an effective therapeutic regimen or alternative options.

## 1. Introduction

Nocardiosis is a rare bacterial infection caused by gram-positive aerobic* Actinomyces *of the genus* Nocardia* [1, 2]. It is typically regarded as an opportunistic infection that affects mainly patients with cell-mediated disorder, such as acquired immunodeficiency syndrome (AIDS) patients or those with solid tumors and hematologic malignancies, those with transplant recipients, and those on prolonged glucocorticoid therapy [2].* Nocardia *spp. are ubiquitous soil organisms that are aerosolized with dust and respiratory tract is the main portal of entry. Organisms can also be acquired by direct inoculation resulting in primary infections of the skin and subcutaneous tissues [2]. Infections in immunocompetent host (about one-third) [1] are typically chronic processes localized to a single organ or region; in contrast, hematogenous dissemination, frequently involving central nervous system, skin, and soft tissues, is characteristic of immunocompromised hosts [2].

There are no pathognomonic signs or symptoms of nocardiosis and clinical picture may be mistaken for a variety of other bacterial infections including actinomycosis, tuberculosis, fungal infections, and malignancies [2]. A definitive diagnosis requires the isolation and identification of the organism, but cultures of* Nocardia *spp. grow slowly (ranging from 2 days to several weeks) and are difficult to obtain. Although it grows on ordinary blood agar plates and does not require special breeding methods, laboratory should always be informed when nocardiosis is suspected because the diagnosis may be missed by routine methods. Susceptibility testing is particularly important as different species and strains often have markedly different susceptibility patterns. Gram stain and modified acid-fast stain must be considered for the initial evaluation [3]. Serology is usually not useful, as no single serological technique can detect all of the clinically relevant species. Molecular technics such as 16S rRNA sequencing, polymerase chain reaction (PCR), and real-time PCR provide accurate and rapid results, but this technics are not available in most clinical laboratories [2].

The most appropriate therapy administration route and treatment duration have not yet been well established in clinical trials. Sulfonamides have been considered the mainstay of therapy, and a combined regimen consisting of a sulfonamide, amikacin, and either a carbapenem or a third-generation cephalosporin has been proposed for high-risk patients [3, 4].

No specific aspects of clinical presentation, diagnosis, and treatment of nocardiosis are described for HIV infected patients. This case report illustrates some difficulties in the diagnosis of this condition and the response to a different treatment regimen than what is suggested in the literature.

## 2. Case Report

A 46-year-old Guinea-Bissau born male, living in Portugal for 16 years, was admitted to our institution because of vomiting, hiccups, weight loss (about 16 kg), occasional nocturnal sweating, and dry cough, lasting for 1 month. He had a past medical history of recurrent episodes of intestinal worm infection and excessive alcohol consumption (100–120 g/day). He was screened for tuberculosis due to prior contact with pulmonar tuberculosis. Laboratory blood tests were normal except for a high sedimentation rate (79 mmh). A bilateral bronchial reinforcement was observed on chest X-ray. Mediastinal enlarged lymph nodes and a nodular lesion at inferior left pulmonary lobe were noted on chest CT (computerized tomography) scan. A brain CT showed a small corticosubcortical ring-enhancing lesion in the right frontal lobe. He was diagnosed with HIV-1 infection (323/8,3% T-CD_4_^+^ lymphocytes/*μ*L) and HBV chronic infection (AgHBs positive, Ac anti-HBc positive, and AgHBe negative).

**Figure 1:**
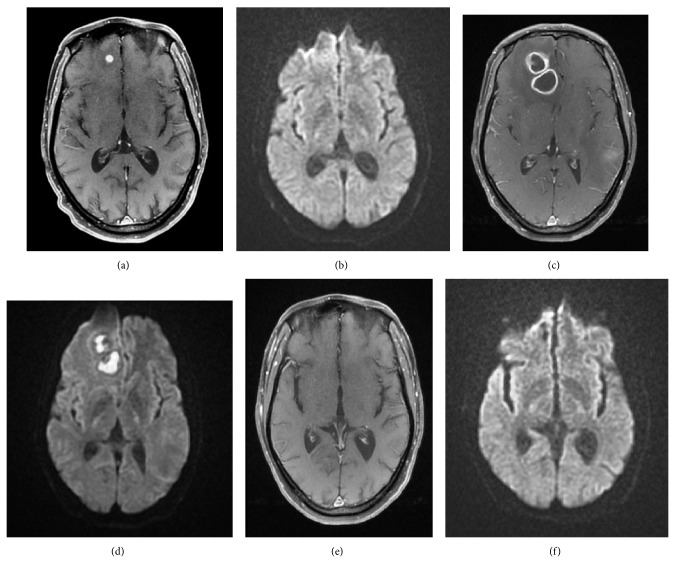
Single small nodular enhancing lesion with discrete central hyposignal (a) not seen on DWI (b). Two ring enhancing lesions (c), with partial restricted diffusion (d). No lesions seen on T1 WI post gad (e) and DWI (f).

Inspection revealed good general condition, with papular lesions scattered throughout the body. On brain MRI a single round lesion with 7 mm was seen, with perilesional oedema, suggesting tuberculoma (Figures 1(a)-1(b)).

Cerebrospinal fluid (CSF) cytochemical evaluation was normal. Bronchoscopy showed nonspecific inflammatory findings. Bacteriological and mycobacteriological (direct and cultural examination) analysis of respiratory products tested negative. Bronchial histology showed inflammatory infiltrate, with no granuloma or neoplastic tissue. Patient was discharged from hospital clinically stable, with no signs of infection.

One month later he manifested persistent deterioration of general condition, with vomiting, abdominal pain in the right upper quadrant, myalgias, and fever. Laboratory tests revealed leukocytosis, C-reactive protein 12,2 mg/dL, and sedimentation rate >120 mmh. On chest CT scan a bulky right adrenal gland mass was disclosed. Abdominal sepsis was considered and treatment with intravenous (IV) ceftriaxone (1 g bid) was empirically started. Blood, urine, and stool cultural exams were negative, as well as* Schistosoma *spp. and* Taenia solium *serology. He completed a 14-day cycle of antibiotic treatment with clinical and analytical improvement. He was also treated with oral fluconazole (200 mg id) during 14 days for esophageal candidiasis. One week after ceftriaxone suspension a worsening of headache and inaugural seizures were observed. MRI brain-scan presented multiple well-defined annular lesions.* Nocardia* versusfungal abscesses was hypothesized (Figures 1(c)-1(d)). Empirical treatment with IV ceftriaxone (2 g bid), liposomal B amphotericin (4 mg/Kg id), and valproic acid was started. Adrenal mass was punctured; culture of purulent material revealed* Nocardia *spp. Characterization of bacterial species and antimicrobial susceptibility testing were not possible, neither by microbiologic methods nor by protein chain reaction (the sample was deteriorated when it arrived at reference laboratory; in Portugal, characterization of* Nocardia* spp. and antimicrobial susceptibility testing are made at reference laboratory, National Health Institute, Dr. Ricardo Jorge). ACTH and cortisol were within normal limits. As skin nodules persisted, a skin biopsy was performed; histology revealed superficial acute folliculitis and no microorganisms were seen with special stains; microbiological study was missed. The diagnosis of disseminated nocardiosis with brain, adrenal, and possibly lung and skin involvement was assumed. Based on literature, IV treatment was started with cotrimoxazole 1440 mg 8/8 h, amikacin (dosage adjusted to the serum levels), and ceftriaxone (2 g bid); liposomal B amphotericin was stopped.

Two months later the patient showed clinical and laboratory improvement. His CD_4_^+^ cell count was then 207 (7,1%)/*μ*L and viral load was 491640 cp/mL (genotypic resistance test was negative); HBV viral load was 777873717 UI/mL. An antiretroviral scheme with HBV activity was initiated with lopinavir/ritonavir (LPV/RTV) and tenofovir/emtricitabine (TDF/FTC). Four weeks later there was deterioration of renal function (GFR 69,2 mL/min). Amikacin was stopped. As renal failure went on worsening (GFR 37,7 mL/min), TDF/FTC was replaced by abacavir/lamivudine (ABC/3TC) and then cotrimoxazole was also stopped. Attempting to maintain a sulfonamide on the antibiotic regimen, dapsone was introduced. A significant transaminases elevation (AST 513 U/L; ALT 622 U/L with normal bilirubin) was interpreted as hepatotoxicity and dapsone was discontinued. The possibility of reactivation of HBV infection regarding the replacement of TDF/FTC for ABC/3TC was also considered. HBV viral load was still detectable (782 UI/mL), but at a lower level, so entecavir was added. Treatment with IV ceftriaxone (2 g bid) was kept for 1 year. Reassessment with Brain-MRI showed remaining lesions (Figures 1(e)-1(f)) and abdominal-CT scan showed resolution of adrenal abscess; HIV viral load was on the threshold of detectability (54 cp/mL) as well as HVB viral load (16 UI/mL).

Currently, six months after stopping ceftriaxone, the patient is asymptomatic and presents a good recovery of renal function (GFR 80 mL/min). He is under valproic acid (800 mg bid) and antiretrovirals, with virologic suppression of HIV and HBV and improvement of immune status (306/12% T-CD_4_^+^ lymphocytes/*μ*L).

## 3. Discussion 

This is a report of a disseminated nocardiosis as primary opportunistic disease in an AIDS patient. It is a rare condition in HIV infected patients, affecting those with advanced immunosuppression (CD_4_^+^ < 200/*μ*L) [2]. Low prevalence of disease is possibly due to diagnostic difficulties. Routine use of cotrimoxazole as prophylaxis for* Pneumocystis jiroveci *pneumonia or toxoplasmosis in severely immunocompromised patients may have also a protective effect against nocardial infection. On the other hand, identification of acid-fast bacilli on direct examination can be mistaken with* Mycobacterium* spp., which is a main opportunistic disease affecting HIV infected patients [5]. Finally, it is also a result of earlier introduction of antiretroviral treatment [2]. Lung seems to be the organ most frequently involved presenting as localized infection or as primary site for dissemination.* N. asteroids* are the main cause of disseminated infection, followed by* N. brasiliensis* [5].

Four sites of infection were involved in this case: adrenal abscess (microbiological and imaging criterion); multiple central nervous system abscesses (imaging criterion); bilateral bronchial reinforcement and a left pulmonary lobe nodular lesion (imaging criterion); and skin papules (clinical criterion). All these manifestations of infection responded to instituted therapy.

Concerning treatment, sulfonamides are the agents of choice, but on monotherapy they have been associated with frequent relapses and a higher mortality rate. A combination of three drugs, including sulfas, is recommended in patients with severe central nervous system involvement or disseminated disease [2]. In this case, an empiric IV antibiotic scheme with cotrimoxazole, amikacin, and ceftriaxone was initiated. After 2,5 months of treatment, renal toxicity conditioned suspension of cotrimoxazole and amikacin. IV ceftriaxone was maintained for 1 year, according to clinical and imaging resolution of the lesions. The differentiation of* Nocardia *species and the susceptibility testing would have been particularly important to guide alternative treatment options.

There is no data on when to start antiretroviral therapy. In this case we started it 2 months after initiating treatment for nocardiosis, when clinical situation was stable. There were no signs of immune reconstitution syndrome. As the patient compliance was not known, a high genetic barrier scheme was initiated with a protease inhibitor associated with TDF/FTC. TDF was replaced by ABC for nephrotoxicity and entecavir was added to cover HBV infection.

In conclusion, this report highlights some difficulties in management of disseminated nocardiosis in a patient with HIV infection, including the diagnosis and treatment guidance, aggravated by the lack of antibiotic susceptibility test, the incapacity to identify species, and the intolerance to several drugs.
